# Photoluminescence Enhancement of Poly(3-methylthiophene) Nanowires upon Length Variable DNA Hybridization

**DOI:** 10.3390/polym10010100

**Published:** 2018-01-20

**Authors:** Jingyuan Huang, Jinho Choi, Gil Sun Lee, Fengchun Chen, Chunzhi Cui, Long Yi Jin, Dong Hyuk Park

**Affiliations:** 1 Department of Chemistry, College of Science, and Key Laboratory for Organism Resources of the Changbai Mountain and Functional Molecules, Ministry of Education, Yanbian University, Yanji 133002, China; 2154014461@ybu.edu.cn (J.H.); 2015010396@ybu.edu.cn (F.C.); 2 Department of Applied Organic Materials Engineering, Inha University, Incheon 402-751, Korea; jinho@inha.edu; 3 Department of General Education, Kookmin University, Seoul 02707, Korea; gslee@kookmin.ac.kr

**Keywords:** poly(3-methylthiophene), nanowire, DNA length, nanoscale photoluminescence

## Abstract

The use of low-dimensional inorganic or organic nanomaterials has advantages for DNA and protein recognition due to their sensitivity, accuracy, and physical size matching. In this research, poly(3-methylthiophene) (P3MT) nanowires (NWs) are electrochemically prepared with dopant followed by functionalization with probe DNA (pDNA) sequence through electrostatic interaction. Various lengths of pDNA sequences (10-, 20- and 30-mer) are conjugated to the P3MT NWs respectively followed with hybridization with their complementary target DNA (tDNA) sequences. The nanoscale photoluminescence (PL) properties of the P3MT NWs are studied throughout the whole process at solid state. In addition, the correlation between the PL enhancement and the double helix DNA with various lengths is demonstrated.

## 1. Introduction

The effective conjugation of biological materials with functional condensed matters plays an important role in biological recognition in terms of accuracy, sensitivity, and signal response time [1,2,3,4,5]. Organic π-conjugated materials such as polythiophene and polydiacetylene have been considered as active systems for biological recognition due to their compatibility with biological molecules and their properties of optical and electrical signal [6,7,8,9,10,11]. As an alternative strategy, a simple and effective method of optically-direct (i.e., without fluorescent dye) DNA detection can be proposed by the light-emitting polymer nanowires (NWs) with lightly-doped states [12]. These polymers possess negative counter-ions with dipole characteristics from dopants placed near (much less than 10 nm) the cationic polymer chains [13]. The dopants can introduce effective energy transfer due to dipole interactions and easily attach with biomaterials, such as DNA and proteins through electroactive interactions [14,15,16]. However, most studies of π-conjugated polymer based-biorecognition have shown their usefulness in aqueous states [17,18,19]. In previous research, we performed target DNA (tDNA) recognition by doped poly(3-methylthiophene) (P3MT) single NW, and the dopant was successfully conjugated the probe DNA (pDNA) [20]. In addition, to recognize other important biomolecules, protein, we also utilized an aptamer funtionalized single P3MT NW [21]. Raman signal enhancement of the NW was achieved due to the resonance effect between the wavelength of the excitation laser and absorption of phase transitioned NW. Moreover, the photoluminescence (PL) intensity was also augmented owing to the enhanced crystallinity of the P3MT polymer chain upon recognition of protein. Alternatively, the use of inorganic materials such as silicon NWs to detect DNA by measuring its conductivity has been reported [22,23]. In particular, conductivities upon sequence variations of tDNA or hybridization sites of tDNA to peptide nucleic acid (PNA) were also measured. However, as we know, a similar identification study has not been performed using organic nano-materials.

In this study, light-emitting P3MT NWs were electrochemically fabricated using dodecylbenzne sulfonic acid (DBSA) as a dopant based on an anodic alumina oxide (Al_2_O_3_) template [24]. The pDNA was conjugated easily to the P3MT NWs through electrostatic interactions between the sulfuric trioxide (SO_3_^−^) from the dopant and the terminal amine (NH_3_^+^) from pDNA. We selected three various lengths of pDNA sequences (10-, 20- and 30-mer) and attached them on the surface of the P3MT NWs, respectively. Color change (green to red) of the single P3MT NW was observed after attaching the pDNA. Then, we applied the corresponding complementary tDNA sequences on the P3MT NWs, respectively. The effect of DNA hybridization upon sequence length on P3MT NW was observed by PL signal and mapping analysis at nanoscale. Finally, conversion of the dominant peak in the PL spectra upon length variable DNA hybridization was also discussed.

## 2. Materials and Methods

The light-emitting P3MT NWs were electrochemically fabricated using dodecylbenzne sulfonic acid (DBSA) dopant through an anodic alumina oxide (Al_2_O_3_) template. The Al_2_O_3_ templates (pore diameter of ~200 nm and thickness of 60 μm) were purchased from Whatman Co. (Leicestershire, England). The electrolyte used for preparation of the P3MT NWs consisted of 3-methylthiophene (3MT) as a monomer, tetrabutylammonium trifluoromethan sulfonic acid (TBACF_3_SO_3_) as a dopant, and acetonitrile (CH_3_CN) as a solvent. The molar ratio of monomer to dopant was 5:1. After preparation of the P3MT NWs, a 2 M of HF or 2 M of NaOH solution was used to dissolve the Al_2_O_3_ templates.

The formation of the P3MT NWs was visualized by transmission electron microscope (TEM) (JEOL, JEM-2010, Boston, MA, USA) The luminescence color charge-coupled devices (CCD) images of the NWs were measured using an AVT Marlin F-033C (λ_ex_ = 435 nm). The exposure time of the light was fixed at 1 s to compare the brightness of the CCD images under the same experimental conditions. The solid (i.e., under dry conditions) PL images and spectra for an isolated single NW strand were measured using a laser confocal microscopy (LCM) built around an inverted optical microscope (Axiovert 200, Zeiss GmbH, Jena, Germany). The 488 nm line of an unpolarized argon ion laser was used for LCM PL excitation. The spot size of the focused laser beam was estimated to be approximately 190 nm. The incident laser power and the acquisition time for each PL spectra were fixed in the LCM PL measurements at 1 μW and 1 s, respectively.

DNA sequences were synthesized from Bioneer Co. (Dae-jeon, Korea). Probe and complementary target DNA sequences were as follows.

10-mer DNA sequences:
  pDNA: NH_2_-GAG AGA GAG A
  tDNA: CTC TCT CTC T
20-mer DNA sequences:
  pDNA: NH_2_-GAG AGA GAG AGA GAG AGA GA
  tDNA: CTC TCT CTC TCT CTC TCT CT
30-mer DNA sequences:
  pDNA: NH_2_-GAG AGA GAG AGA GAG AGA GAG AGA GAG AGA
  tDNA: CTC TCT CTC TCT CTC TCT CTC TCT CTC TCT

To couple the pDNA with the P3MT NWs, an NH_2_ group was attached to the 5′ of the pDNA sequence. The pDNA was diluted with deionized (DI) water (with the initial resistivity of 18 MΩ·cm Milli-Q water purifier) to a concentration of 100 nM. The immobilization of pDNA onto P3MT NWs was performed for 10 min with constant stirring (~900 rpm). The resulting pDNA-functionalized P3MT NWs were dropped onto the cover glass substrate then the solvent was evaporated. The concentration of the tDNA dissolved in PBS buffer (pH = 7.4) was 100 nM and 100 μL of the solution was dropped on to the substrate. After incubation in a humid chamber for 10 min, the substrate was lightly rinsed with PBS buffer. All samples were dried in a vacuum oven for 30 min to allow measurement solid-state optical spectra under dry conditions.

## 3. Results

**Figure 1:**
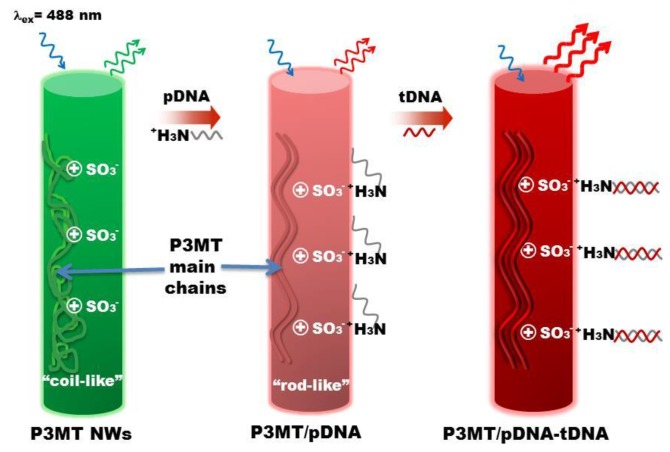
Schematic illustration for optical tDNA recognition using light-emitting P3MT NWs.

A conceptual illustration of enhanced PL efficiency upon tDNA recognition is shown in Figure 1. Upon coupling with pDNA through electrostatic interaction, the luminescence color of the P3MT NWs varies from green to red, which originated from a modification of polymer chain conformation through coupling pDNA. The enhancement of PL signal for the P3MT NWs upon recognition of tDNA is due to the transition of chain configuration.

**Figure 2:**
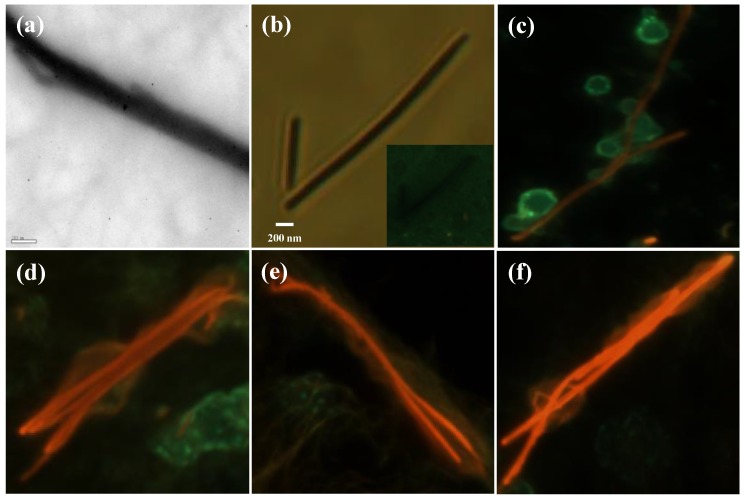
(**a**) TEM image and (**b**) optical image of as-prepared P3MT NW. Charge-coupled devices (CCD) images of (**c**) P3MT/pDNA (10-mer), (**d**) P3MT/pDNA-tDNA (10–10 pairs), (**e**) P3MT/pDNA-tDNA (20–20 pairs) and (**f**) P3MT/pDNA-tDNA (30–30 pairs).

Figure 2a,b shows the TEM and optical images of as-prepared single strand of P3MT NW, respectively. It can be observed by the microscopic analysis that the P3MT NW has a length of several tens of micrometers and a diameter of ~200 nm. In addition, in the luminescent color CCD image, the luminescence color of the P3MT NW is green with weak brightness as shown in the inset of Figure 2b. After being conjugated with pDNA, the color of the P3MT NW changes from green to red (Figure 2c). In this case, we chose the 10-mer single strand pDNA sequence. The luminescence color change of the P3MT NW through coupling with pDNA is owing to conformational modification of the P3MT main chains by electrostatic interactions between SO_3_^−^ from the dopant and the NH_3_^+^ from pDNA as well as the repulsive interaction between SO_3_^−^ and negative charged phosphate backbone of pDNA. However, it is worth noting that DNA hybridization is induced the enhancement of the luminescence intensity of the P3MT NWs compared to that of the pDNA conjugated P3MT NWs as shown in Figure 2d. A similar phenomenon has been reported in previous literatures, both in aqueous and solid state [18,19,20]. It is considered that, after interacting with target molecules as DNA or protein, the P3MT NWs adopted the more plannar rod-like configuration with more region-regular like characteristics [19,21]. Then the relationship between the hybridized double helix DNA (20–20 and 30–30 pairs) and the luminescent intensities was studied. Obviously, luminescence intensities of the case of 20–20 and 30–30 pairs are gradually enhanced compared with the case of 10–10 pairs, as shown in Figure 2e,f, respectively. However, we were unable to confirm quantitative comparison of the luminescent intensities between the three kinds of P3MT NWs.

**Figure 3:**
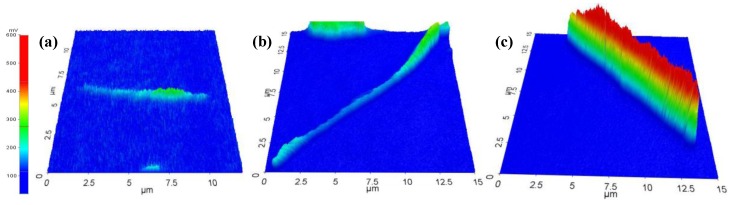
3-D photoluminescence (PL) images of (**a**) P3MT/pDNA-tDNA (10–10 pairs), (**b**) P3MT/pDNA-tDNA (20–20 pairs) and (**c**) P3MT/pDNA-tDNA (30–30 pairs) single NWs. The color scale bar on the left-hand side represents the photon counts.

Hence, a quantifiable analysis upon three-dimensional (3-D) LCM PL images of the P3MT NWs was taken under the same LCM experimental conditions. Figure 3 shows the 3-D LCM PL images of the single P3MT NW which were subjected to the DNA hybridization of 10–10, 20–20, and 30–30 pairs, respectively. The average voltages of the LCM PL intensities for the corresponding case were 215 (±5), 235 (±5) and 420 (±5) mV, respectively. The quantitative comparison based on the 3-D LCM PL images of the single P3MT NWs is in good agreement with the result of the CCD images. Nonetheless, a comprehensive and statistical analysis of the luminescent enhancement is still needed.

**Figure 4:**
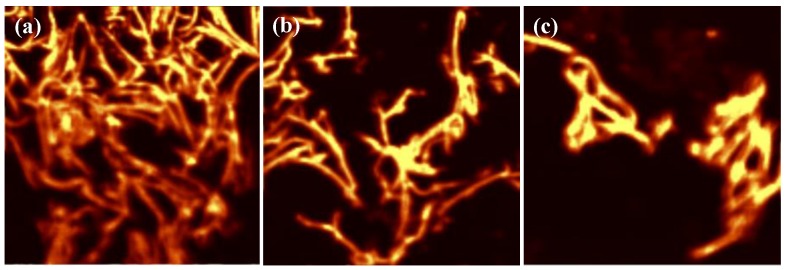
Laser confocal microscopy (LCM) PL mapping images of (**a**) P3MT/pDNA-tDNA (10–10 pairs), (**b**) P3MT/pDNA-tDNA (20–20 pairs) and (**c**) P3MT/pDNA-tDNA (30–30 pairs) NWs, upon excitation with 488-nm laser.

For more in-depth analysis, an LCM PL mapping experiment was conducted under the same experimental conditions. The PL mapping experiment was conducted by using a high-resolution confocal scanning microscope combined spectrometer and diode laser with wavelength of 488 nm. The spatial distribution of the instrument permits mapping for statistical analysis of the P3MT NWs at nanoscale. For PL mapping, the spectral range covered the 500–1000 nm region. The mapping image could be obtained by determining the relative intensities of the PL peak bands. As shown in Figure 4, it is evident that the P3MT NWs treated by 30–30 pairs (Figure 4c) present more intense PL signals than the case of 10–10 and 20–20 pairs. In addition, the calculated maximum PL intensities of the P3MT NWs were 2100 (10–10 pairs), 2550 (20–20 pairs) and 2950 (30–30 pairs), respectively. Although we consider that the PL intensities of the P3MT NWs are also proportional to the longer hybridized double helix DNA (more than 30 pairs), the limitation of the DNA length needs to be demonstrated in the future work. Nonetheless, compared with the other analyses such as surface resonance (SPR) imaging sensor [25,26], organic π-conjugated materials based mapping analysis provides another approach for biomolecules recognition.

**Figure 5:**
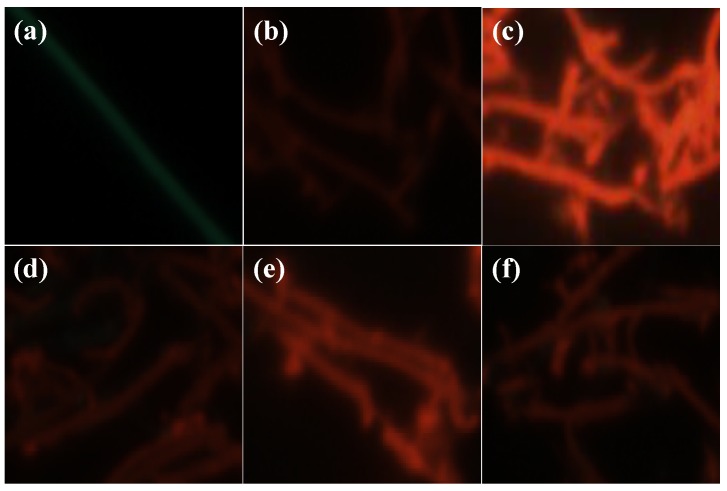
CCD images of (**a**) pristine P3MT, (**b**) P3MT/pDNA (30 pair), (**c**) P3MT/pDNA-tDNA (30–30 pairs), (**d**) PBS buffer treated P3MT/pDNA (30 pair), (**e**) 1-mer mismatched tDNA treated P3MT/pDNA (30 pair) and (**f**) thrombin protein treated P3MT/pDNA (30 pair), respectively.

To confirm that the PL enhancement is induced by the hybridization between pDNA and its complementary tDNA, we also performed a control experiment using PBS buffer solution, a 1-mer mismatched tDNA sequence and a thrombin protein. Figure 5a–c shows the CCD images of the pristine P3MT, P3MT/pDNA (30 pair) and P3MT/pDNA-tDNA (30–30 pairs), respectively. When the P3MT/pDNA (30 pair) NWs were immersed by their complementary sequences, the enhancement of the PL was clearly observed. However, as the P3MT/pDNA (30 pair) is immersed by PBS buffer solution, 1-mer mismatched tDNA and thrombin protein, the P3MT/pDNA (30 pair) NWs still emit weak PL signal, as shown in Figure 5b,d,e, respectively. Hence, it can be considered that the P3MT/pDNA system possesses excellent target selectivity and previous research has also proved this [20,21].

**Figure 6:**
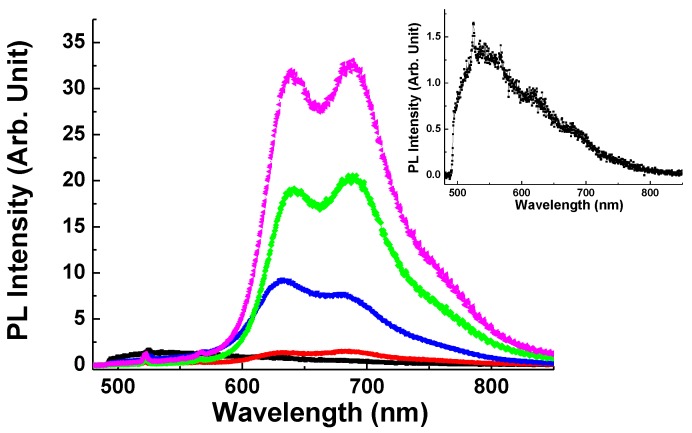
LCM PL spectra of doped P3MT (black line), P3MT/pDNA (red line), P3MT/pDNA-tDNA (10–10 pairs) (blue line), P3MT/pDNA-tDNA (20–20 pairs) (green line) and P3MT/pDNA-tDNA (30–30 pairs) (pink line) NWs. Inset: Magnified LCM PL spectra of doped P3MT NWs.

Finally, the average PL spectra of each sample were also taken from corresponding mapping images as shown in Figure 6. The main PL peak for the pristine P3MT NWs detected at ~554 nm (i.e., green emission) as shown in the inset. It is the result of an intra-chain event, and is associated with a non-planner coil-like conformation [27]. Shoulder peaks at longer wavelengths are due to planarization and inter-chain events due to contact between P3MT chains [28]. After conjugation with pDNA, it induced a redshift of the peak at ~544 nm to ~645 and ~680 nm (i.e., red emission). According to the literature, the emission peaks of ~645 and ~680 nm are contributed from an inter-chain event [28,29]. This means that the pDNA forces the coil like backbone of P3MT NWs to become stretched. Furthermore, it shows that the PL intensities of the P3MT NWs increase with the hybridized DNA length. This suggests that the local planarization of the P3MT backbone will lead to increased contact between nearby polymer chains and we consider that this increased contact is proportional to the length of hybridized DNA [19]. In addition, it is interesting to note the conversion of dominant peak upon hybridized DNA length. It can be observed that, as the P3MT NWs are subjected to the DNA hybridization of short length (10–10 pairs), the dominant PL peak is at ~645 nm. However, as the P3MT NWs subjected to the cases of 20–20 and 30–30 pairs, the dominant PL peak shifts to ~680 nm. Obviously, the intrinsic PL properties of the P3MT NWs affected by the length of the hybridized double helix DNA sequences. A detailed understanding of how these factors affect the intrinsic PL properties of the P3MT NWs is presently lacking and these issues should be addressed in our future work.

## 4. Conclusions

In summary, P3MT NWs are electrochemically prepared by the assistance of dopant. As-prepared NWs are conjugated with various lengths of pDNAs followed by hybridization with their complementary tDNAs, respectively. Through the analyses of color CCD images, 3-D PL images, mapping images and PL spectra of the P3MT NWs, it was found that the PL intensities of the NWs are proportional to the length of the hybridized DNA. With the results of this research, we look forward to providing a new avenue for detailed bio-recognition as identification of mismatch position in DNA sequence or species of target materials.
